# Abstract of the Proceedings of the Buffalo Medical Association

**Published:** 1858-02

**Authors:** Austin Flint


ART. VI.—*Abstract of the Proceedings of the Buffalo Medical Association.*

TUESDAY EVENING, Jan. 5, 1857.

The association met.

Present—The Vice-President, Dr. Wyckoff, in the chair. Drs. Root, Nichols, White, Mixer, Strong, Hunt, Samo, Newman, Lemon, Gay, Nott, Treat, Flint, Jr., Rochester, Rogers, Whitney, Ring, Devening, and Garvin.

A reading of the minutes of the last meeting was called for, but as they were not present, it was dispensed with.

*Prof. Hunt* remarked that as he would not be able to be present during the whole of the proceedings, it would, perhaps, be better to appoint a secretary pro. tem.

On motion, Dr. Flint, Jr., was appointed secretary pro. tem.

The committee on puerperal convulsions, asked further time to report, which was granted.

*Prof. White* presented an ovarian tumor which was sent to him by Dr. Ackley, of Cleveland. When it was first removed it measured thirty-six inches in its largest circumference, but it was now somewhat diminished in size. The following is a description of the case which was furnished by Mr. Garlick, an advanced student, who was present at the operation.

Miss ——, æt. 34 years, came under care of Dr. A., having a tumor of large size situated in the abdomen, and which was evidently ovarian. Patient was in excellent health, and there was no evidence of malignant disease. Although informed of the severe nature of the operation, she desired its removal, which was accordingly done six weeks ago to-day.

Patient having been placed on a table, and all things put in readiness, she was brought under the influence of chloroform to insensibility; an incision was then made from umbilicus to symphysis pubis in a direct line. There was a very considerable amount of adipose tissue to be gone through before reaching the peritoneum—fully two and a-half to three inches. The peritoneum being divided to the whole extent of the first incision, the tumor presented itself in plain view; an opening of small size was then made in the tumor, and a thick glairy fluid flowed rapidly from it, until by this means it became reduced in size, and by a sudden movement of the patient, was forced out through the incision, so that by its own bulk, it prevented any of the fluid from getting within the abdominal parietes. After all, or nearly all, of the fluid had come away, the tumor was found to be attached by a small pedicle, about which a very strong ligature was placed, and the tumor removed at its neck. In closing the wound a few sutures were used, and the ligature which had been placed about the pedicle of the tumor, was brought forward and fastened by one of the sutures at the lower part of the incision.

Calomel and opium were given to the patient, as symptoms seemed to indicate, for the next six days, at the end of which time her bowels were moved, but not before. The calomel was then discontinued, but small doses of opium were still occasionally given.

Patient did not suffer a great amount of pain. Considerable pus was evolved, apparently from the incision, but this discharge at length ceased, and in four weeks from the time of operation, the patient was able to sit up in her room for a while each day, and was rapidly convalescing.

The tumor removed, measured thirty-six inches by its longest circumference, but has since shrunk four or five inches.*

This is the sixth case which has been operated upon by Dr. Ackley, in four of which he has been successful, and in two, unsuccessful.

When this subject was brought up at a former meeting of the association, Dr. White was not in favor of the operation, and conceived that the chief objection lay in the great difficulty of diagnosis; in the first place the dis-

---

* The letter of Prof. Ackley to Dr. White, was handed to one of the editors of the Journal, but was mislaid, and cannot be found; the notes of the case by Mr. Garlick, and the description by Dr. White, embody all the facts of the case, though we regret that we are unable to present the original letter from Dr. Ackley.

crimination between ovarian disease and intra-uterine tumor; and again between the unilocular and multilocular variety of tumor. Mr. Clay, of Manchester, has made mistakes in the diagnosis of this disease, and after opening the abdominal cavity, has found that there was no tumor of the ovary. Ordinary surgeons cannot pretend to greater skill than Mr. Clay, and it would be an extremely mortifying and embarrassing thing for a surgeon to make the operation and find that he had been mistaken in diagnosis.

The question now arises, what can we do in such cases? Remedies, it is well known, are inefficacious; we may employ sorbefacients and diuretics, but the fluid cannot be removed, because the lining membrane of the walls are not supplied with absorbents. We have, then, two alternatives, namely, tapping and removal. As all therapeutical measures are useless, we cannot dismiss this operation entirely. A cure may be effected by the production of inflammatory action. Dr. White related a case where inflammation followed injury inflicted by a fall upon the abdomen. In this case the diagnosis was doubtful; a tumor occupied the abdomen, and the patient met with a severe fall, striking upon the abdomen; peritonitis followed, and she was permanently cured. This was effected in the same way, as a bursa is cured by a sharp blow, thereby causing a rupture of the walls, with escape and absorbtion of the contained fluid. The simple operation of tapping is not entirely without danger, two out of ten cases being followed by a fatal result. We are unable to determine whether the tumor consists of one, or many cysts, and in performing this operation we must be guided in a great measure by this circumstance, as in tapping a multilocular tumor we do very little good, being able to open only one of the cysts, and the others gradually enlarging to fill up the space. A mode of treatment adopted by some, is the injection of stimulating fluids into the cavity and the excitating of inflammation, in the same manner as we cure an effusion of fluid into the vaginal tunic of the testicle in hydrocele.

The statistics which Dr. W. has now at his command, show eighty-nine cases of extirpation, thirty-four of which proved fatal. The members will probably be surprised, in looking at the statistics of other operations, which are admitted to be perfectly justifiable, to find how large a proportion of cases terminate fatally. All operations are attended with some danger. Out of 853 amputations reported by Malgaigne, including even, amputations of fingers and toes, 332 died, making a proportion of about four in ten, about the same mortality as in the operation under consideration. In cases of amputation of the thigh, we have a mortality of 7 in 10. In 554 operations for hernia, 260 died, making a proportion of 7 in 10. If it were not therefore, for the great difficulty in diagnosis, this operation would seem to rank with operations about which we have no doubt as to their safety and justifiability. There is another method of treatment which has not been mentioned, and which consists in the establishment of a fistulous opening into the peritoneal cavity, so that the fluid may come into contact with the absorbents with which this membrane is so richly endowed, and be thus removed.

*Dr. Flint, Jr.*, presented specimens of scales of bone which he removed from the posterior part of the falx cerebri, on the inner surface of the dura mater. These specimens were examined by him under the microscope, and found to consist of true bone.

Rokitansky mentions these formations as being quite common, and usually found in the situation from which this specimen was removed. They are ordinarily considered to be attached to the dura mater, but Rokitansky thinks that they are attached to the parietal arachnoid.

*Prof. Hunt* presented a table for medical and surgical statistics, from the Medical Society of the State of New York. It is intended that a copy of this table shall be placed in the hands of every regular physician in the state, for the registration of disease, beginning from the first of January. At the end of the year, this record should be returned to the society, or to the secretary of the State society. The table is accompanied by a full set of explanations and directions, and will be the means of arriving at most useful results. Prof. Hunt also presented a number of valuable pamphlets to the library of the association, which were received, and a vote of thanks passed.

On motion, Drs. Strong, of Chatauque Co., and Orton, of London, England, were invited to participate in the discussions, etc., of the association.

*Prof. White* presented to the association two letters from Dr. Congdon, which were accompanied by two interesting specimens.

JACKSONVILLE, TOMPKINS CO., N. Y.,
December 22d, 1857.

DR. WHITE,

Dear Sir: You will find enclosed a pathological specimen, of which the following is a brief history:

Miss ——, aged 40, of a large stout frame, and of a wealthy and esteemed family, says that she has been troubled for twenty years with vaginal leucorrhœa, attended, she has been told by physicians, with retroversion of the uterus, says that she has suffered much from "bearing down pains," so much so that at times she has not been able to walk about her room. Has been "regular" most of the time, till within the last year, in which she has been quite "irregular." Has generally suffered much pain during menstruation. Has had some severe fits of sickness: "liver complaint," of course, has been one of her most prominent ailments. She has been treated by various physicians and quacks, sometimes being benefited and sometimes injured.

I saw and prescribed for her for the first time, Nov. 25, 1857, at which time she was suffering from all the symptoms attending both vaginal and uterine leucorrhœa; walked about her room but little, and that with the greatest care. Being naturally of a plethoric habit, her appearance was not extremely anæmic.

I prescribed citrate of iron dissolved in a decoction of some of the vegetable tonics and an injection of arg. nit., grs. xx— ℥i, to be used every third day, with a ℥iv glass syringe.

The patient being some eight miles from me, I did not see her again until Dec. 4th, at which time she says the discharge from the vagina was very large the first time she used the arg. nit., and resembled curdled milk, among which she found strips of membrane; says she feels better.

Made no alteration in the prescription, excepting same mild laxative, and the arg. nit. to be used gr. x—ʒi, with injections into the vagina of tepid water, once or twice a day.

Dec. 10th. Says she feels much as after my last visit, and shows me a pathological specimen similar to the one I send you, which she says passed the vagina on using an injection.

Prescription continued.

Saw her again to-day, Dec. 23d. She reports better, and shows me three such specimens as I send you, and says that she has passed some six or seven in all, four or five of them being perfect, the others more or less torn.

The specimen I send you, when first passed, would hold water, but its walls have adhered in drying. In some of them the walls are much thicker. The paper to which this specimen adheres, is exactly the shape of the specimen, so if it should get broken before it reaches you, you will have its shape. She says the day before passing these she has pain, similar to labor pain, I should judge by her description.

Such specimens may be common, but I do not think that physicians often see them. I have seen portions of such membrane before, but never any so perfect.

I wish that you would exhibit it before your medical association, that I, as well as the rest of the profession may be benefited by your discussions, through the Buffalo Medical Journal.

Yours in haste,

L. CONGDON.

JACKSONVILLE, Dec. 31, 1857.

PROF. WHITE,

Dear Sir: * * * * That you may be better acquainted with the case, I send you its history for the past week. I visited her yesterday; says she feels better; has thrown off two such membranes as I sent you, during the past week, making some eight or ten that have been thrown off in about thirty or thirty-five days, (I intend to obtain the exact data of each.)

This case, it appears to me, plainly disproves the respective positions taken by Professors Simpson and Deman on this subject. Dr. Churchil says, *probably* Dr. Locock is right in *supposing* it to be the result of an inflammation of a *peculiar* kind, and concludes by admitting that *probably* he (Churchil) do n't know much about it.

I much regret that there is so little positive testimony recorded in relation to the pathology and treatment of such cases. There may be more recorded than I am aware of, but all authors that I now have access to, are quite speculative on this subject. I am sure the subject is of sufficient importance to demand much observation and research, for there is no greater scourge undermining the health of our females than those diseases that pertain to the uterine organs.

Please find within another specimen presented me to-day by another patient whom I have been treating the past year for dysmenorrhœa and leucorrhœa. This patient, married, aged 26, a farmer's daughter, quiet in her habits and of high respectability, never borne children, has generally been healthy, with the exception of the dysmenorrhœa, which has always been quite troublesome, and more recently very serious, acccompanied, or rather preceded and followed by leucorrhœal discharges.

She has frequently shown me shreds and pieces of membrane during the past year, but has never discovered anything perfect like the one I send you until to-day.

In both of the above cases the size of membrane would indicate an enlargement of the uterus, which is undoubtedly the case in the first, but the symptoms in the second are not so indicative.

Both these cases are now under the following treatment: Daily ablutions of warm water, internal use of citrate of iron, iodide of potassium, and mild laxatives. Injections of nitrate of silver and tannic acid, sufficient to keep the vaginal leucorrhœa in check. * * *

Respectfully yours,

L. CONGDON.

P. S. The within membrane first, thrown off, was perfect even to the cervix uteri. L. C.

*Prof. White* remarked that it is now the generally received opinion, that the membrane which we find frequently, if not always, thrown off in dysmenorrhœa, is identical with the deciduous membrane of pregnancy. The old idea, that this membrane was formed by a plastic exudation on the internal surface of the uterus, is now entirely abandoned by the most reliable authorities on this subject, and it is now fully conceded that it is produced by a hypertrophy of the mucous membrane of the uterus, which takes place at every menstrual period. When the ovum is fecundated, this hypertrophy, instead of ceasing at the expiration of the menstrual function, goes on, and forms the decidua vera, offshoots of which enclose the ovum and form the decidua reflexa. By reference to the letter of Dr. Congdon, it will be seen that membranes like the first specimen, were thrown off about every five days.

Dr. White is of the opinion that there is a form of inflammation of this organ, which is attended by a formation of false membrane, like the membrane of true croup, which is formed and thrown off repeatedly. From the fact that this membrane is thrown off at such short intervals, it would seem that it was of that character. A portion of the first specimen was given to Dr. Nichols, but he only subjected it to a cursory ond hurried examination, and from his investigation was not able to satisfy himself of the presence of any of the prominent characteristics of mucous membrane. The second specimen, he had no opportunity of examining. The second specimen, on the other hand, is thrown off during the menstrual perion, and is accompanied by dysmenorrhœa, which would indicate that it is a true decidua, cast off and regenerated in the manner previously described. The fact of this periodical loss of mucous membrane which the uterus sustains, more particularly at parturition, probably induced some of the older anatomists to hold the opinion that this organ was not supplied with mucous membrane; as at this time the muscular fibres are entirely denuded. These specimens are of great interest, and Dr. White takes great pleasure in being able to present them to the society.

[These specimens have been subjected to further examination in the following manner, and with the following results: A small portion of each was put into a slightly saline solution in different vessels, and allowed to remain for twenty hours. The fluid consisted of water with a little salt, merely enough to give a saline taste. At the end of twenty hours, a small portion of the first specimen was removed from the solution and placed between two plates of glass. In doing this, a portion of the membrane was carefully spread out, and a small portion gently torn by dissecting needles; this was then moistened by the fluid in which it had been soaked, and subjected to examination with a magnifying power of 284 diameters (Nachet's large instrument.) On bringing the object into focus, a large quantity of pavement epithelial scales were seen, which were exceedingly distinct and beautiful. These were observed detached and isolated in that portion of the specimen where the membrane had been lacerated by the dissecting needles. On removing the field to this portion where the membrane had not been disturbed it was found to be entirely covered by the polygonal plates of tesselated epithelium; no trace of the fibrillation of plastic matter was observed in any portion of the specimen; thus conclusively proving that it consisted of an exfoliation of true mucous membrane. This specimen was somewhat discolored, probably by the injections of nitrate of silver which had been made use of during the treatment of the case.

The second membrane was then examined in the same way, and found to consist of the same materials. The tesselated epithelial scales were here even more beautifully apparent, as this specimen was free from discoloration.

These examinations were made in the presence of, and confirmed by Drs. White, Flint, Jr., Nichols and Orton, and Mr. Mason.

In examining the first specimen, which was thrown off every fourth or fifth day, we did not expect to find the evidences of mucous membrane, as it seemed improbable that it could be regenerated and cast off at such short intervals. It was on this supposition as well as the non-appearance of epithelial scales, on the first superficial examination, that Prof. White conceived that this was an illustration of his theory of dyphtheritic inflammation of the uterus. The facts which are disclosed by the microscope, however, do not disprove this theory, but they merely show that this particular case is not an example. If we had found the appearances of coagulated fibrin, with a few epithelial scales, we might have supposed that the appearance of epithelium was merely accidental, and that it had been brought away with the exudation; but this was not the case; all that appeared under the microscope, was epithelium, and epithelium in situ, (that is, applied to its basement membrane in layers, and formed into polygonal plates by mutual compression;) with the basement membrane. The appearances, indeed, were conceded by all present, to be a beautiful example, and such as it is rarely our good fortune to see, of the anatomy and mode of application of the epithelial covering of mucous membrane.

In the examination of the second specimen, the microscope disclosed precisely what we expected to find. This you will recollect was thrown off during menstruation, and was accompanied by dysmenorrhœa; and in such cases we generally, if not always, have an exfoliation of the proper mucous coat of the uterus. F.]

*Dr. Wyckoff* then remarked, that by the records of the Boston Society for Medical Improvement, as published in the Boston Medical and Surgical Journal, a few weeks since, he saw the propriety of operating for the relief of imperforate anus and rectum is called in question; and it is even urged by some of the members that it is better that a child should die than that an operation should be attempted. Successful cases have since been reported, however, through the same Journal. He would also submit a case of

*Imperforate Rectum; Operation successful.*

Mrs. R., of Buffalo, was delivered, June 15, 1857, of a plump healthy female child; labor natural and easy.

June 16. I was informed by the nurse that the child had had no evacuation from the bowels. I ordered injections, but found that they were not retained, but were followed by violent straining, which produced redness of the face and vomiting of yellow matter. When upon introducing first the probe and then the finger, I found the rectum obstructed about an inch from the external surface, by a firm membrane that protruded downward by the forcing efforts of the child.

June 17. Dr. Hamilton in consultation. After examining the case, an operation was decided upon, and Dr. H. operated by carrying a sharp pointed bistoury upon the finger to the membrane and cutting it freely from before backwards; whereupon an immense passage of meconium followed. The opening was farther enlarged by making a crucial incision, and passing the index finger oiled. On finding it impracticable to retain a bougie or anything of the kind to prevent contraction while healing, it was determined to leave it alone until the third day, when the index finger, oiled, was passed, and broke up the adhesions already formed; this gave the child much pain, and was followed by painful passages; the passage grew more and more painful and smaller, util the 29th of June, when there was no pássage at all.

June 30th. Upon an examination with the finger I found the passage closed, when I passed a probe pointed bistoury and separated the parts freely, laterally and posteriorly; the child was again relieved by passing large fœcal stools.

The child grew as fast and seemed to be doing as well as other children of its age, until the 12th of July, when the stools were observed to be smaller and frequent, accompanied by great tenesmus and crying; the abdomen became much swollen and tender, which continued with all the symptoms growing worse daily, until

July 18th. When Dr. Hamilton again operated by cutting the parts freely, both laterally and posteriorly, which gave the child perfect relief.

No unpleasant symptoms followed the last operation.

Dec. 7th. Examined the rectum by passing the finger, and found a thin membrane about three-quarters of an inch from the surface, through which the finger would pass readily. The child is in perfect health and has no difficult whatever in passing fœces.

*Prof. White* remarked, that the case reported by Dr. Wyckoff was certainly a suitable one for an operation. He had seen two cases operated upon by Paul Dubois, one of which terminated fatally. He had seen another case in this city, which was under the charge of a German practitioner. Dr. White was consulted, and as soon as he was aware of the nature of the case, advised an immediate operation; the child expired, however, on its way to the office. Dr. W. cannot conceive that it is justifiable to allow an infant to perish without giving it the chance of life which will result from an operation.

*Dr. Strong*, of Chatauque Co., said that he had read the discussions which appeared in the Buffalo Medical Journal, in reference to fracture of the skull accompanied by hæmorrhage from the ear. Four cases had come under his observation, of hæmorrhage from the ear, as the result of a blow. In three of these cases, the hæmorrhage occurred from both ears; and in one, from only one ear. All of them recovered. One of these cases was accompanied by strabismus, which continued for two days, and was followed by double vision, lasting for a few days more. In these cases the Dr. was not able to detect any fracture, and as they all terminated in recovery, the occurrence of hæmorrhage from the ear is certainly not a fatal sign.

*Dr. Strong*, of Buffalo, asked the opinion of the association in relation to the operation of tracheotomy in cases of true croup. It seemed to him that the hands of the profession were tied in these cases, and that there was nothing for the physician to do, but to look on and see his patient pass out of the world. It certainly seems as though something should be done, but the opinion of the profession is so uniformly opposed to the operation, that it is an act of the greatest responsibility to attempt it. Some surgeons, indeed, uniformly discountenance it and will perform it under no circumstances whatever. In a case which would be inevitably fatal, without an operation, it would appear that the operation of tracheotomy would be justifiable, as giving the patient some slight chance of life. Dr. S. had a case of true croup last summer, where the father of the child was much distressed because the Dr. would not consent to perform the operation.

*Prof. Rochester* remarked that it would be well to refer this subject to a committee.

*Prof. White* was not aware that this operation was so generally discountenanced, as was implied by Dr. Strong. He conceived that it was generally opposed by parents, until it was too late. Trousseau has operated in more than 100 cases, in one half of which the operation was successful. Dr. White had seen him operate upon two cases. One of the great reasons why he thinks that the operation is not more frequently made, is that the parents are usually unwilling; another reason is, that the good results which have followed the application of nitrate of silver, have rendered the use of this measure so common, that it has kept off the operation until it is too late.

*Dr. Strong* replied that the cases which demanded an operation, were in young children, of from one to two years old: the case which he mentioned was a child four years of age.

Some discussion then arose concerning the application of nitrate of silver to the laryngeal mucous membrane. Dr. White thought that there was no difficulty in introducing the probang, if the finger be used as a guide, thus conducting the sponge to the rima glottidis. Another method is to seize the tongue by means of a dry linen cloth, and pull it forward.

*Prof. White* moved that a committee of one be appointed to report on this subject, which was carried.

The chair appointed Dr. White, who declined. The chair then appointed Dr. Rochester.

*Dr. Strong* wished to know the sentiments of the members of the association in regard to the dinner of the Erie Co. Society, whether it would be thought advisable to omit it for this year. This suggestion met with no responce, and it was concluded that the members were willing to have the dinner as usual.

The treasurer, Dr. Newman, suggested that the rent would soon become due, and that there would not be sufficient funds in the treasury to meet it. He stated that many members were in arrears for dues.

*Prof. Hunt* stated that extra copies of the transactions of the association could be worked off at very small cost when they were set up for the Journal, and that it might be desirable that these should be collected and bound for the use of the association and its members.

No action was taken on this proposition.

The association then adjourned.

AUSTIN FLINT, Jr., M. D.,
Secretary *pro tem.*

